# Coincubation as miR-Loading Strategy to Improve the Anti-Tumor Effect of Stem Cell-Derived EVs

**DOI:** 10.3390/pharmaceutics13010076

**Published:** 2021-01-08

**Authors:** Alessia Brossa, Marta Tapparo, Valentina Fonsato, Elli Papadimitriou, Michela Delena, Giovanni Camussi, Benedetta Bussolati

**Affiliations:** 1 Department of Molecular Biotechnology and Health Sciences, University of Torino, 10126 Torino, Italy; alessia.brossa@unito.it (A.B.); elli.papadimitriou@unito.it (E.P.); 2 Molecular Biotechnology Center, University of Torino, 10126 Torino, Italy; marta.tapparo@unito.it (M.T.); valentina.fonsato@2i3t.it (V.F.); michela.delena@gmail.com (M.D.); 3 Department of Medical Science, University of Torino, 10126 Torino, Italy; giovanni.camussi@unito.it; 4 Society for Business Incubator and Tech Transfer, University of Torino, 10126 Torino, Italy

**Keywords:** extracellular vesicle engineering, microRNA, loading, anti-tumor, cancer stem cells, exosomes, coincubation

## Abstract

Extracellular vesicles are considered a novel therapeutic tool, due to their ability to transfer their cargoes to target cells. Different strategies to directly load extracellular vesicles with RNA species have been proposed. Electroporation has been used for the loading of non-active vesicles; however, the engineering of vesicles already carrying a therapeutically active cargo is still under investigation. Here, we set up a coincubation method to increase the anti-tumor effect of extracellular vesicles isolated from human liver stem cells (HLSC-EVs). Using the coincubation protocol, vesicles were loaded with the anti-tumor miRNA-145, and their effect was evaluated on renal cancer stem cell invasion. Loaded HLSC-EVs maintained their integrity and miR transfer ability. Loaded miR-145, but not miR-145 alone, was protected by RNAse digestion, possibly due to its binding to RNA-binding proteins on HLSC-EV surface, such as Annexin A2. Moreover, miR-145 coincubated HLSC-EVs were more effective in inhibiting the invasive properties of cancer stem cells, in comparison to naïve vesicles. The protocol reported here exploits a well described property of extracellular vesicles to bind nucleic acids on their surface and protect them from degradation, in order to obtain an effective miRNA loading, thus increasing the activity of therapeutically active naïve extracellular vesicles.

## 1. Introduction

Extracellular vesicles (EVs) are nanosized vesicles actively released by many, if not all, cells and identified in biological fluids [1]. EVs display the ability to deliver active cargo, including RNA species, to target cells, thus reprogramming their gene expression profile [1]. Therefore, the interest for the exploitation of EVs as therapeutic tool is rapidly increasing [2]. Indeed, EVs appear a highly efficient delivery system, as compared to naked molecules, as they protect the cargo from degradation by RNases and proteases [3,4]. Moreover, in comparison to synthetic liposomes, EVs might display an increased efficacy since, being a natural cell derived product, they show reduced clearance by the macrophagic system, low immunogenicity and ability to deliver nucleic-acid-based therapeutics across biological barriers [5,6]. 

A great deal of effort has been, therefore, dedicated to the pharmacological exploitation of EVs as carriers of specific cargo of interest [7]. In particular, EV engineering with RNA species has been proposed for different therapeutic applications, ranging from anti-tumor strategies to vaccination [4,8,9]. Different technical approaches for EV loading include the option to modify the originating cell, typically by transfection, or to directly load isolated EVs using electroporation [4]. The approach of direct EV loading might be of particular interest in those contexts where the delivery of the identified cargo represents the main mechanism of action of the therapeutic preparation. For instance, we recently set up a methodology for direct electroporation of EVs isolated from plasma for the loading of antitumor microRNAs without perturbation of their integrity and targeting properties [10]. In this context, the use of plasma-derived EVs from an autologous source might appear of therapeutic relevance. 

A different situation can be envisaged when EVs carrying per se a therapeutically active cargo are loaded with a desired molecule to increase their effect. We recently showed that EVs from human liver stem cells (HLSC) induced a potent anti-tumor effect in vitro and in vivo [11,12,13,14]. Specifically, HLSC-EVs inhibited the invasion of renal cancer stem cells (rCSCs) [14], and induced their apoptosis [12]. Preliminary experiments attempting to load HLSC-EVs with an additional miR cargo through electroporation not only failed to increase their therapeutic effect, but rather reduced it, suggesting the loss of their endogenous activity.

The aim of the present paper was, therefore, to identify a strategy to potentiate the endogenous anti-tumor activity of stem-cell derived EVs avoiding the loss of their endogenous effect. For this purpose, exploiting the EV ability to bind, protect and transport active RNA species on their surface, we set up a coincubation protocol able to load microRNAs on HLSC-EVs and to increase their anti-tumor effects. 

## 2. Materials and Methods

### 2.1. Renal Cancer Stem Cells Isolation and Culture

Renal cancer stem cells (rCSCs) were isolated and characterized as previously described [12,14,15]. Cells were obtained from specimens of renal cell carcinomas from patients undergoing nephrectomy, according to the Ethics Committee of the S. Giovanni Battista Hospital of Torino, Italy (168/2014, 16 August 2014). CD105-positive rCSCs were isolated by magnetic cell sorting from the total tumor cell population, using the magnetic-activated cell sorting (MACS) system (Miltenyi Biotec, Auburn, CA, USA). Single CD105 positive cells were seeded in 96-well plates in presence of the expansion medium, consisting of DMEM LG (Invitrogen, Carlsbad, CA, USA), supplemented with 2 nM l-glutamine (Lonza, Basel, Switzerland), insulin-transferrin-selenium, 10-9 M dexamethasone, 100 U penicillin, 1000 U streptomycin, 10 ng/mL epidermal growth factor (EGF) (all from Sigma-Aldrich, St. Louis, MO, USA) and 5% fetal calf serum (FCS) (Euroclone, Pero MI, Italy). A CD105 positive clonal rCSC line was selected and used for all the experiments, as previously described [12,14]. Mycoplasma absence was routinely tested using RT-PCR.

### 2.2. Human Liver Stem Cells Isolation and Culture

Human Liver Stem Cells (HLSCs) were generated by Anemocyte International (Gerenzano, Italy) from a liver donor, according to the standard criteria of Centro Nazionale Trapianti, as previously described [14,16]. Isolated HLSCs were cultured in the presence of minimal essential medium (α-MEM; Lonza, Basel, Switzerland) supplemented with 10% FCS (Euroclone), 10 ng/mL human recombinant EGF (Miltenyi, Bergisch Gladbach, Germany), 10 ng/mL human recombinant basic fibroblast growth factor (Miltenyi, Bergisch Gladbach, Germany), 2 nM l-glutamine (Lonza, Basel, Switzerland) and 100 U/mL penicillin/streptomycin (Sigma, St. Louis, MO, USA) and maintained in a humidified 5% CO_2_ incubator at 37 °C. After 2 weeks, HLSC colonies were expanded and characterized as previously described [16]. Mycoplasma absence was routinely tested using RT-PCR.

### 2.3. HLSC-EVs Isolation and Characterization

For EV isolation, sub-confluent HLSCs were cultured overnight in serum-free α-MEM (Lonza, Basel, Switzerland); the supernatant was then recovered and centrifuged for 20 min at 3000 *g* before being filtered (0.22 µm filters, Merck-Millipore, Burlington, MA, USA) in order to remove cell debris and apoptotic bodies. Supernatants were then ultracentrifuged (Beckman Coulter Optima L-90 K, Fullerton, CA, USA) at 100,000 *g* for 2 h at 4 °C. HLSC-EVs were resuspended in RPMI supplemented with 1% dimethyl sulfoxide (DMSO, Sigma-Aldrich, St. Louis, MO, USA) and stored at −80 °C for later use. Nanosight LS300 system (Malvern Panalytical, Malvern, UK) was used to evaluate EVs concentration and size distribution. Briefly, EV preparations were diluted (1:200) in sterile saline solution and analyzed by the Nanoparticle Analysis System using the NTA 1.4 Analytical Software, as previously described [12,14].

### 2.4. Electroporation Protocol

HLSC-EVs were electroporated using Invitrogen Neon Kit (Invitrogen, Carlsbad, CA, USA), as previously described [10]. Briefly, 6 × 10^10^ EVs were electroporated with a voltage of 750 V using a pulse width of 20 ms, for 10 pulses, according to the manufacturer’s protocol. EVs were then was incubated for 30 min at 37 °C, washed by ultracentrifugation at 100,000 *g* for 2 h at 4 °C, resuspended in RPMI (Lonza, Basel, Switzerland) and immediately used for selected experiments. For each experiment, 6 × 10^10^ HLSC-EVs were subjected to 37 °C incubation and ultracentrifugation without electroporation to be used as control.

### 2.5. Coincubation Protocol

EVs (10^10^) were incubated for 1 h at 37 °C with 100 picomoles of the indicated miRNA, in a final volume of 200 µL RPMI (Lonza, Basel, Switzerland). When indicated, coincubation was followed by RNAse-A (LifeTechnologies, Carlsbad, CA, USA) treatment (0.1 ng/μL) for 3 h at 37 °C to digest free miRNA. RNAse digestion was stopped by incubation with 4U of RNAse inhibitor (Invitrogen, Carlsbad, CA, USA) for 1 h at 37 °C. When indicated, RNAse treatment was followed with trypsin digestion (5 ng/mL, 1 h at 37 °C) (Sigma-Aldrich, St. Louis, MO, USA). Samples were then subjected to centrifugation (4000 RPM, 5 min at 4 °C) using 50 kDa filters (Merck-Millipore, Burlington, MA, USA), in order to remove unbound and undigested miRNAs, and immediately used for indicated experiments.

### 2.6. Cytofluorimetric EV Analysis

HLSC-EVs were bound to surfactant-free white aldehyde/sulfate latex beads 4% w/v, 4 μm diameter (Molecular Probes, Thermo Fisher, Waltham, MA, USA) for the cytofluorimetric analysis using Guava instrument (Merck-Millipore, Burlington, MA, USA). Thirty μg of EVs were incubated with 5 μL of beads for 30 min at room temperature and subsequently for 30 min at +4 °C. Adsorbed EVs were then incubated with FITC and PE labeled antibodies against CD63, CD44, integrin alpha 4 and CD29 (all from Beckton Dickinson, Franklin Lakes, NJ, USA) with a final dilution of 1:50, for 15 min at +4 °C. During the cytofluorimetric acquisition, the gating strategy was set on the physical parameters dot plot. Controls corresponded to EVs adsorbed on beads and marked with FITC-, PE- or APC-conjugated mouse IgG1 Isotypes (all from Miltenyi, Bergisch Gladbach, Germany). 

### 2.7. Apoptosis

Cytofluorimetric evaluation of apoptotic cells was performed using the Muse™ Annexin V & Dead Cell Kit (Merck-Millipore, Burlington, MA, USA), according to the manufacturer’s instructions. Briefly, 10 × 10^3^ cells were incubated with 50 × 10^3^ EVs/target cell for 48 h. Cells were then detached and resuspended in Muse™ Annexin V & Dead Cell Kit (Luminex, Austin, TX, USA), and the percentage of apoptotic cells (Annexin V^+^) was detected.

### 2.8. Invasion

Invasion assay was performed using 24-well cell culture inserts (Beckton Dickinson, Franklin Lakes, NJ, USA) with a porous membrane (8.0 μm pore size) precoated with 100 μg growth factor-reduced Matrigel (Beckton Dickinson, Franklin Lakes, NJ, USA) per well, as previously described [14]. Briefly, 50 × 10^3^ rCSCs were detached using a non-enzymatic solution (Sigma-Aldrich, St. Louis, MO, USA), and plated in the presence of 50 × 10^3^ EVs/target cell in the upper side of the pre-coated transwell in DMEM (Euroclone). As an attractive stimulus, complete culture medium was added in the well. Every condition was performed in triplicate. After 48 h, cells that moved from the upper side of the transwell to the lower one were fixed in MetOH and stained with crystal violet (Sigma-Aldrich, St. Louis, MO, USA). The total area of invaded Matrigel (original magnification: 100×) was evaluated by ImageJ on at least five pictures per transwell.

### 2.9. Super-Resolution Microscopy

Super-resolution analyses were performed using a Nanoimager S Mark II microscope from ONI (Oxford Nanoimaging, Oxford, UK) equipped with 405 nm/150 mW, 473 nm/1 W, 560 nm/1 W, 640 nm/1 W lasers and dual emission channels split at 640 nm. For the preparation of the sample, 10 μL of Poly-l-Lysine (Sigma-Aldrich, St. Louis, MO, USA) was placed on coverslips, in culture wells (Grace Bio-Labs, Sigma-Aldrich, St. Louis, MO, USA), and left at 37 °C in a humidifying chamber for two hours. After removal of the excess, HLSC-EVs previously coincubated with a Scrambled-FITC RNA sequence and digested with RNase, were left to attach overnight at +4 °C on the coverslips. The next day, non-attached EVs were removed and 10 μL of blocking buffer (PBS-5% Bovine Serum Albumin) was added into the wells for 30 min. Then, 2.5 µg of purified mouse anti-CD29 antibody (Beckton Dickinson, Franklin Lakes, NJ, USA) were conjugated with Alexa Fluor 647 dye, using the Apex Antibody Labeling Kit (Invitrogen, Carlsbad, CA, USA) according to the manufacturer’s protocol. Anti-CD29 Alexa Fluor 647 antibody was added to the blocking buffer containing wells at a final dilution 1:10. The antibody was left for overnight incubation at +4 °C. The samples were washed twice with PBS and 10 μL ONI BCubed Imaging Buffer was added for the EV imaging. Two-channel dSTORM data were acquired sequentially at 30 Hz (Hertz) in total reflection fluorescence (TIRF) mode. Single molecule data was filtered using NimOS (Version 1.7.1.10213, ONI, Oxford, UK) based on the point spread function shape, photon count and localization precision to minimize background noise and remove low-precision localizations.

### 2.10. EV Incorporation in Target Cells

To evaluate the internalization of coincubated EVs in rCSCs by fluorescent microscopy, HLSC-EVs were labeled with 1 μM Dil dye (ThermoFisher, Waltham, MA, USA) as described previously [12]. Briefly, HLSC-EVs were resuspended in PBS supplemented with 1 μM Dil dye and ultracentrifuged at 100,000 *g* for 1 h at 4 °C. EVs were then washed with PBS by ultracentrifugation (100,000 *g* for 1 h at 4 °C). The EV pellet was resuspended in RPMI and processed for coincubation. Coincubated EVs were immediately used to treat previously plated rCSCs (50 × 10^3^ EVs/target cell) for 1 h, cells were then fixed in 4% paraformaldehyde (Sigma-Aldrich, St. Louis, MO, USA) and processed for confocal microscopy.

### 2.11. miRNA Isolation and Real Time PCR

Total RNA was isolated from different rCSCs or EVs preparations using MirVana kit (Ambion, ThermoFisher, Waltham, MA, USA), according to the manufacturer’s protocol, and quantified spectrophotometrically (Nanodrop ND-1000, ThermoFisher, Waltham, MA, USA). First-strand cDNA was produced from 200 ng of total RNA using the miScript Reverse Transcription Kit (Qiagen, Hilden, Germany). Real-time PCR experiments were performed in 20 µL reaction mixture containing 5 ng of cDNA template, the sequence-specific oligonucleotide primers (purchased from MWG-Biotech, Nantes, BRU, Luxembourg) and the miScript SYBR Green PCR Kit (Qiagen, Hilden, Germany). RNU48 was used to normalize miRNA inputs. 

### 2.12. Protein Extraction and Western Blot

Different preparations of HLSC-EVs were lysed in RIPA buffer supplemented with protease and phosphatase inhibitor cocktail and PMSF (Sigma-Aldrich, St. Louis, MO, USA) immediately after ultracentrifuge. Aliquots of EV lysates containing 30 μg proteins, as determined by Bradford quantification (Biorad, Hercules, CA, USA), were run on 4–20% SDS-PAGE under reducing conditions and blotted onto PVDF membrane filters using the iBLOT system (LifeTechnologies, Carlsbad, CA, USA). Membranes were blocked in Tris-buffered saline-Tween (TBS-T; 25 mM Tris, pH 8.0, 150 mM NaCl, and 0.05% Tween-20) containing 5% (*w*/*v*) non-fat dried milk for 1 h. After blocking, membranes were incubated overnight with anti-ANAXA2 antibody (LS-C150122, LSBio, Seattle, WA, USA). Blots were then incubated with Goat anti-Rabbit IgG HRP conjugated (Thermo Scientific, Waltham, MA, USA) for 1 h at room temperature. Membranes were then probed with ClarityTM Western ECL substrate (Bio-rad, Hercules, CA, USA), and bands were detected by the Chemidoc system (Bio-rad, Hercules, CA, USA).

### 2.13. Statistical Analysis

Statistical analysis was carried out on Graph Pad Prism version 5.04 (GraphPad Software, Inc, San Diego, CA, USA) by using the Student t-test or ANOVA with Dunnet’s multi-comparison tests, where appropriate. A *p* value < 0.05 was considered significant.

## 3. Results

### 3.1. Comparison between Electroporation and Coincubation to Increase the EV Antitumor Effect

We previously demonstrated the anti-tumor effect of HLSC-EVs on renal cancer stem cells (rCSCs) [12,13,14]. With the aim of potentiating this effect, we decided to enrich EVs with anti-tumor miRNAs, known to display a strong anti-invasive and pro-apoptotic effect in rCSCs [14]. For this aim, we performed parallel experiments in which we compared EV miRNA loading using electroporation, previously set in our laboratory [10], or coincubation, already reported to be effective [7], on induction of rCSCs apoptosis. 

We first assessed the maintenance of functional properties in EVs after coincubation with a scrambled RNA sequence in comparison with electroporation, in the absence of miRNA loading, by testing their biological activity on rCSCs apoptosis induction [12] (Supplementary Figure S1A). Electroporated EVs showed reduced pro-apoptotic activity, when compared to naïve HLSC-EVs, as already reported [12]. In addition, electroporated EVs were unable to induce the endogenous expression of miR-200a and miR-200b, known to be responsible for the anti-tumor effect of naive EVs [14], suggesting loss of active cargo in electroporated EVs (Supplementary Figure S1B). At variance, EVs coincubated with a scrambled sequence maintained their pro-apoptotic effect (Supplementary Figure S1A), together with the induction of the expression of anti-tumor miRNAs (Supplementary Figure S1B). 

**Figure 1:**
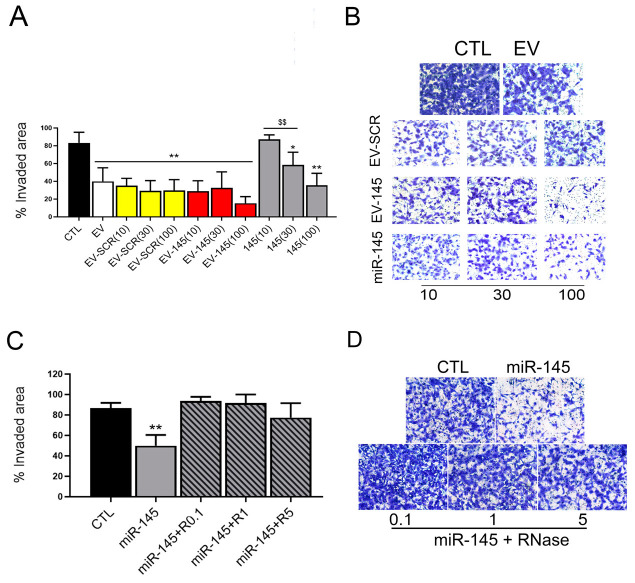
Effect of miR145 coincubated HLSC-EVs on rCSC invasion. (**A**,**B**) Quantification (**A**) and representative micrographs (original magnification: 100×) (**B**) of rCSCs invasion after treatment for 48 h with HLSC-EVs (EV) loaded with different doses (100/30/10 pmol/10^10^ EVs, corresponding to 6000/2000/600 molecules/EV) of a scrambled sequence (EV-SCR) or with miR-145 (EV-145), or miR-145 alone (145); all at a dose of 10, 30 or 100 pmol/10^10^ EVs. Data are represented as mean ± SD of the percentage of invaded area of three experiments. * = *p* < 0.05 and ** = *p* < 0.001 vs. CTL; $$ = *p* < 0.001 vs. 145(100). (**C**,**D**) Invasion assay quantification (**D**) and representative micrographs (original magnification: 100×) (**E**) of rCSCs treated for 48 h with miR-145 untreated (miR-145) or digested with 0.1, 1 or 5 ng/µL of RNase-A (miR-145 + R0.1, 1 or 5, respectively). Data are represented as mean ± SD of the percentage of the invaded area of the three experiments. ** = *p* < 0.001 vs. CTL.

Therefore, we decided to set a coincubation protocol for the direct loading of the anti-tumor miRNA miR-145, which is present at a low level in naïve HLSC-EVs [14]. In particular, we focused on assessing whether miR-145 loading could potentiate the effect of HLSC-EVs on the reduction of the high invasive property of rCSCs [14]. HLSC-EVs incubated with different doses of a scrambled sequence (EV + SCR 100/30/10 picomol, corresponding to 6000/2000/600 miR-molecules/EV) maintained the anti-invasive effect on rCSCs (Figure 1A,B), at levels comparable to naïve EVs. Subsequently, generation of miR-145 loaded EVs by coincubation with miR-145 potentiated the anti-tumor effect of naive EVs in terms of invasion (Figure 1A,B). In particular, different doses of miR-145 tested (EV-145 100/30/10 pmol/10^10^ EVs, corresponding to 6000/2000/600 miR-molecules/EV, red bars), increased the anti-invasive effect of naïve EVs, with the higher dose (100 picomol/10^10^ EVs) being the most efficient. This dose was selected for the following experiments. MiR-145 alone, used as control of unbound miRNA, at the highest dose (100 picomol/10^10^ EVs, corresponding to 6000 miR-145 molecules/EV), also exerted an anti-invasive effect (Figure 1A,B), since miRNA molecules could aggregate during EV engineering, as previously shown [17].

### 3.2. RNAse Treatment of Coincubated EVs

In order to obtain miR-145 coincubated EVs in the absence of unbound miR-145, we took advantage of RNAse treatment. Indeed, incubation of miR-145 alone with 0.1, 1 or 5 μg/mL RNAse abolished the observed anti-invasive effect, indicating its susceptibility to RNAse treatment at all doses (Figure 1C,D). Therefore, we chose the lowest RNAse concentration (0.1 μg/mL) to digest the unbound fraction of miR-loaded EVs.

**Figure 2:**
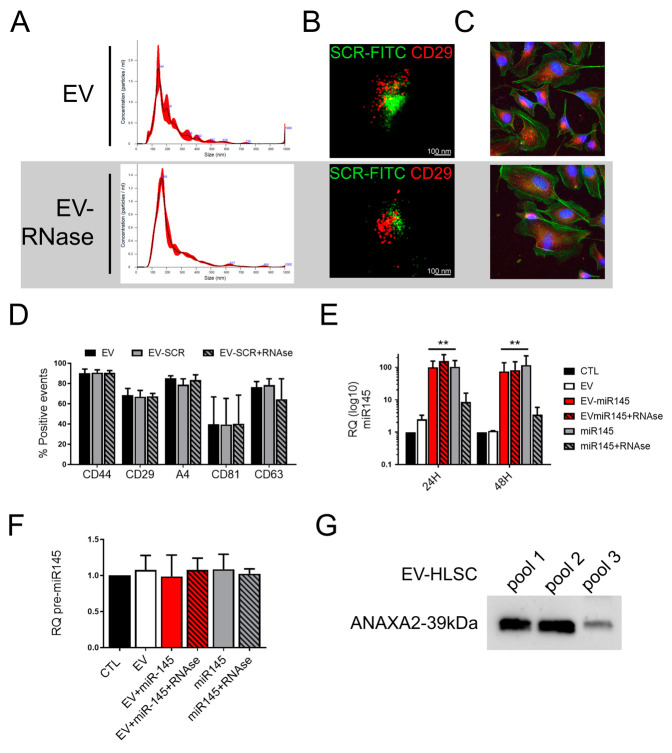
Integrity of miR145 coincubated HLSC-EVs after RNase treatment. (**A**) NanoSight distribution graph showing the quantity and size of HLSC-EVs untreated (Pre-RNase) or digested with 0.1 ng/µL RNase-A (RNase 0.1 ng/µL). (**B**) Super resolution microscopy micrographs showing the effective loading of a scrambled FITC sequence (green). HLSC-EVs are labeled with anti-CD29 Ab (red). Scale bar: 100 nm. (**C**) Representative micrographs of incorporation of DIL-labeled HLSC-EVs (EV) and DIL-labeled HLSC-EVs treated with 0.1 ng/µL RNase-A (EV-RNase) in rCSCs after 1 h of incubation detected by confocal microscopy (original magnification 400×). (**D**) Immunophenotypic characterization of HLSC-EVs (EV) loaded with a scrambled sequence (EV-SCR) and treated with 0.1 ng/µL RNase-A (EV-SCR + RNase), expressing the markers of cells of origin (CD44, CD29 and integrin alpha4 (A4)), together with the exosomal markers CD63 and CD81. Results are mean ± SD of the percentage of positive events of four different independent EV preparations. (**E**) Real Time analysis showing miR-145 levels in rCSCs treated for 24 h or 48 h with naïve HLSC-EVs (EV), HLSC-EVs coincubated with miR-145 untreated (EV-miR145) or digested with 0.1 ng/µL RNase-A (EV-miR145 + RNAse), or with free miR-145 untreated (miR145) or digested with 0.1 ng/µL RNase-A (miR145 + RNAse). Data are represented as mean ± SD of four independent experiments of the Relative Quantification (RQ) normalized to untreated cells (CTL) and to RNU6B. One-way ANOVA was performed: ** = *p* < 0.001 vs. CTL. (**F**) Real Time analysis showing pre-miR-145 levels in rCSCs treated for 24 h with naïve HLSC-EVs (EV), HLSC-EVs coincubated with miR-145 untreated (EV-145) or digested with 0.1 ng/µL RNase-A (EV-145 + RNAse), or with free miR-145 untreated (miR145) or digested with 0.1 ng/µL RNase-A (miR145 + RNAse). Data are represented as mean ± SD of three independent experiments of the Relative Quantification (RQ) normalized to untreated cells (CTL) and to RNU6B. (**G**) Western blot analysis of three different HLSC-EVs preparations (pool1, pool2, pool3) showing the presence of Annexin A2 (ANAXA2).

We first assessed EV integrity after RNAse treatment by NTA (Figure 2A) and super resolution microscopy (Figure 2B). As shown in Figure 2A, the size distribution of coincubated EVs treated with RNAse did not vary with respect to naive EVs. In addition, super resolution microscopy images of HLSC-EVs, coincubated with a FITC-scrambled sequence, confirmed the effective RNA loading and maintenance after RNAse treatment (Figure 2B). Moreover, EV uptake by rCSCs was not affected (Figure 2C). In order to assess whether EV coincubation and/or RNAse treatment could affect EV protein content, we performed flow cytometry analysis on HLSC-EVs, evaluating the percentage of EVs positive for different EV markers (CD44, CD29, integrin alpha4 (A4), CD81, CD63), known to be present on HLSC-EVs. As shown in Figure 2D, we did not detect any change in marker expression, suggesting that EV integrity was maintained.

### 3.3. Anti-Tumor Effect and miR145 Transfer of RNase Treated Coincubated EVs

We applied the above-described protocol of coincubation and digestion of unbound miRNA with RNAse to enrich HLSC-EVs with the anti-tumor miR-145. In order to assess whether engineered HLSC-EVs could transfer miR-145 to target cells, we treated rCSCs with miR-145-loaded EVs (50 × 10^3^/cell) and we analyzed miR-145 levels in rCSCs after 24 and 48 h of EV treatment. As shown in Figure 2E, naïve HLSC-EVs (EV) did not induce in rCSCs any detectable change of miR-145 expression with respect to untreated cells (CTL), while we observed a 100-fold increase in miR-145 levels when rCSCs were incubated with HLSC-EVs loaded with miR-145 (EV-miR145). RNAse treatment did not interfere with miR-145 transfer, since the same increase was observed when coincubated EVs were treated with RNAse (EV-miR145 + RNAse). As suggested by the functional experiments (Figure 1A), miR-145 levels in target cells were also increased by miR-145 alone, but this effect was abolished by RNAse treatment, further confirming RNAse digestion of unbound miRNA (Figure 2E). In order to evaluate if the observed increase of miR-145 in target cells was due to a miR-145 transfer and not to its induction, we analyzed the levels of pre-miR-145 in rCSCs treated with HLSC-EVs coincubated with miR-145 (EV-miR145) or with free miR-145. As shown in Figure 2F, no detectable change in pre-miR145 was observed, confirming miR-145 transfer in rCSCs by HLSC-EVs coincubated with miR-145. At variance, the transfer of free unbound miR-145 levels was reduced after RNAse treatment (miR-145 + RNAse), indicating the degradation of free miR-145 by RNAse (Figure 2E).

### 3.4. Protection of Surface Loaded miRNAs by RNA Binding Proteins

To understand the mechanism of RNAse protection of miR-145 on HLSC-EV surface, we hypothesized the presence of surface RNA-binding proteins that could act to protect bound miRNAs. As shown in Figure 2G, HLSC-EVs expressed Annexin A2 (ANXA2), known to play an active role in miRNA-loading in EVs [18], and to be present on the EV surface [19].

**Figure 3:**
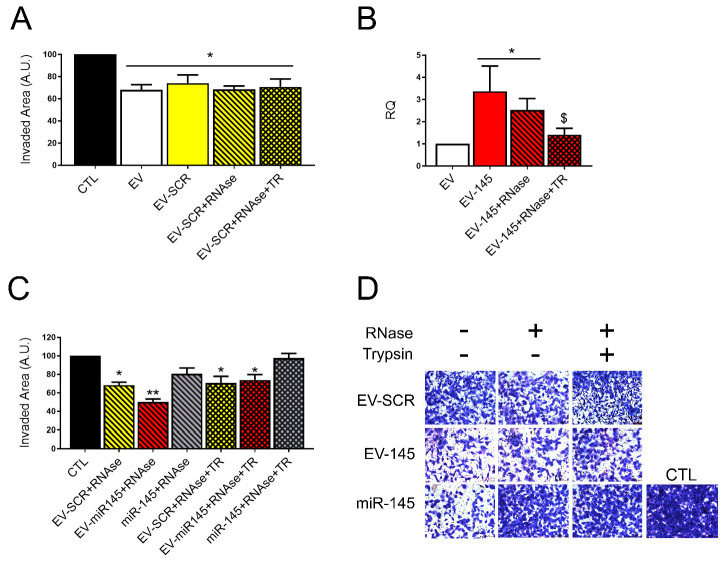
Anti-tumor effect of miR145 coincubated HLSC-EVs after RNase but not trypsin treatment. (**A**) Invasion assay quantification of rCSCs treated for 48 h with naïve HLSC-EVs (EV), or with HLSC-EVs coincubated with a scrambled sequence (EV-SCR), EV-SCR digested with RNase-A (EV-SCR + RNase) and EV-SCR treated with RNase. Data are represented as mean of the percentage of invaded area of one experiment performed in triplicate. (**B**) Real Time analysis showing miR-145 levels in naïve HLSC-EVs (EV), HLSC-EVs coincubated with miR-145 untreated (EV-145) or digested with 0.1 ng/µL RNase-A (EV-145 + RNAse), or digested with RNase and treated with trypsin (EV-145 + RNase + TR). Data are represented as mean ± SD of three independent experiments of the Relative Quantification (RQ) normalized to naïve EVs (EV) and to RNU6B. * = *p* < 0.05 vs. EV and $ = *p* < 0.05 vs. EV-145. (**C**,**D**) Invasion assay quantification (**D**) and representative micrographs (**E**) of rCSCs treated for 48 h with naïve HLSC-EVs (EV), or with HLSC-EVs coincubated with a scrambled sequence or with miR-145 and digested with RNase-A (EV-SCR + RNase and EV-miR145 + RNase, respectively), or with EV-SCR + RNase and EV-miR145 + RNase treated with trypsin (EV-SCR + RNase + TR and EV-miR145 + RNase + TR, respectively). Free miR-145, treated with RNase (miR145 + RNase) or with RNase and trypsin (miR145 + RNase + TR), was used as control. Data are represented as mean of the percentage of invaded area of two experiments performed in triplicate. An ANOVA analysis was performed: * = *p* < 0.05 and ** = *p* < 0.001 vs. untreated rCSCs (CTL).

To further confirm the possible involvement of surface RNA-binding proteins in miRNA loading and protection, we treated EVs with trypsin. As shown in Figure 3A, trypsin treatment did not interfere with EV anti-invasive activity. Therefore, we evaluated the miR-145 levels in HLSC-EVs (EV) and EV coincubated with miR-145 (EV-miR145), which were treated for 3 h with RNAse (EV-145 + RNAse) and for an additional 1 h with trypsin (EV-145 + RNase + TR). As shown in Figure 3B, the enrichment of miR-145 within EVs was high after coincubation and resistant to RNAse treatment but it was significantly decreased after trypsin treatment, further suggesting that miR-145 is bound to RNA-binding proteins present on the EV surface.

In addition, we tested the effect of EVs coincubated with miR-145 on the invasion ability of rCSCs (Figure 3C,D). The effect of naïve EV was increased when EVs were coincubated with miR-145 as expected (EV-miR145). EV treatment with RNAse did not reduce the effect of miR-145 coincubated EVs (EV-miR145 + RNAse), while it reduced that of free miR-145 (miR-145 + RNAse). Trypsin treatment of coincubated EVs (EV-miR145 + TR) reverted the effect of EV-miR145, confirming at a functional level the role of membrane RNA-binding proteins on EV loading with anti-tumor miRNAs by coincubation.

### 3.5. Coincubation Protocol Using Antitumor miRNAs

**Figure 4:**
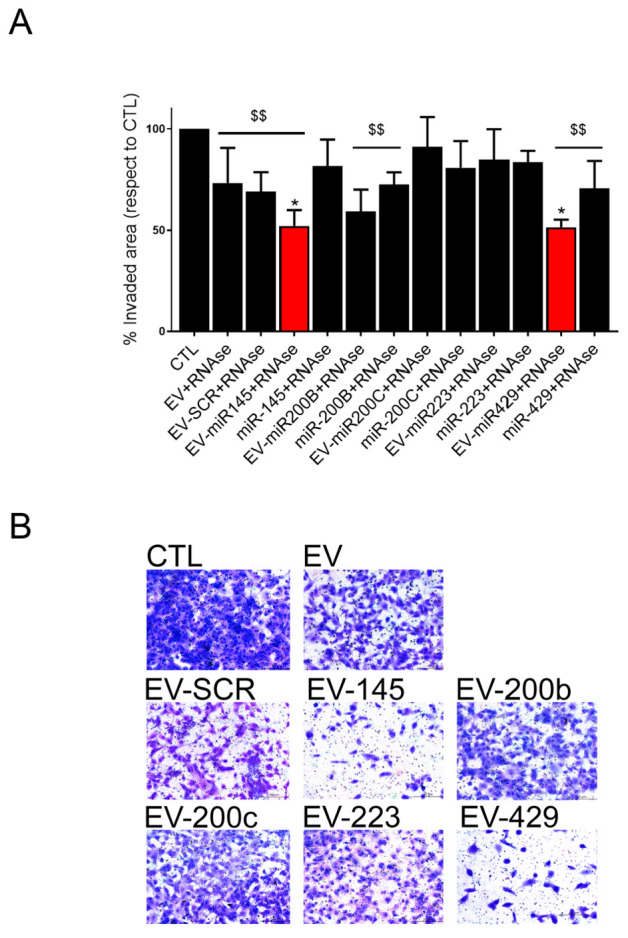
Anti-invasive effects of coincubation invasion assay quantification (**A**) and representative micrographs ((**B**), original magnification: 100×) of rCSCs treated for 48 h with naïve HLSC-EVs (EV), or with HLSC-EVs coincubated with a scrambled sequence or with anti-tumor miRNAs (miR-145, miR200b, miR200c, miR223 and miR429) and digested with RNase-A (EV-miR + RNase). miRNAs alone digested with RNase were used as controls (miR + RNase). Data are represented as mean of the percentage of invaded area of at least two experiments performed in triplicate. An ANOVA analysis was performed: * = *p* < 0.05 vs. EV; $$ = *p* < 0.001 vs. untreated rCSCs (CTL).

Finally, we tested the in vitro effect of HLSC-EVs coincubated with all the anti-tumor miRNAs previously identified as mediators of the anti-tumor effect of HLSC-EVs [14] (miR-145, miR-200b, miR-200c, miR-223 and miR-429). Coincubated EVs were subsequently treated with RNAse in order to exclude any affect due to unbound miRNA. As shown in Figure 4, we observed an additive anti-invasive effect, with respect to naïve EVs, when EVs were coincubated with miR-145 or miR-429.

## 4. Discussion

In the present study, we successfully set up a coincubation protocol able to load microRNAs on HLSC-EVs surface. Engineered co-incubated HLSC-EVs efficiently delivered microRNAs, which were indeed protected by RNase, promoting microRNA-specific functions while maintaining the desired effect of naïve EVs on rCSC. The ability of EVs to bind and transport active RNA and DNA species on their surface is a well-known phenomenon [1,4]. In particular, EVs circulating in serum and present in other biological fluids contain within their corona surface-bound nucleic acids that are considered contaminants. In some cases, additional treatment may be required to remove them from the outside surface of EVs using RNase or DNase [20]. However, membrane-bound RNA species are likely to be protected from RNase degradation, considering the high levels of RNase in biological media such as blood plasma. Indeed, recent reports suggest an active effect of EV surface-associated DNA in horizontal gene transfer of EVs released by mesenchymal stem cells [21,22].

In the present study, we reasoned to exploit the EV ability of binding, protecting and delivering nucleic acids to set up a method for EV engineering. The in vitro assessment of rCSCs invasion was chosen as a readout to compare the effect described for naïve HLSC-EVs [14] with that of engineered HLSC-EVs, in virtue of the simplicity and effectiveness of this test. Considering preliminary experiments showing the loss of biological effect of naïve HLSC-EVs using electroporation, we decided to set up a different protocol able to maintain EV integrity. Indeed, it is conceivable that the electroporation process itself might generate loss of EV membrane integrity with exit of active components, such as RNA species, simultaneously to the entrance of the desired miRNAs. Moreover, several publications have described difficulty in the application of the engineering approach with electroporation because of a high degree of variability, though an effective silencing of the target gene was obtained [23].

To increase the therapeutic effect of HLSC-EVs, we here chose miR-145. In fact, miR-145 is an antitumor miRNA, known to be downregulated in renal cancer [24]. We have recently shown that transfection of rCSCs with miR-145 results in apoptosis induction and tumor cell invasion reduction [14]. Moreover, the transfer of anti-tumor miRNAs to rCSCs, mediated by HLSC-EVs, was able to induce in cancer cells the expression of miR-200 family members, involved in the inhibition of the metastatic process, both in vitro and in vivo [14]. Therefore, the loading of another anti-tumor microRNA, such as miR-145, in HLSC-EVs, resulting in the increase of miR-145 levels in HLSC-EVs, could potentiate the observed anti-tumor effect on rCSCs.

Our protocol successfully preserved the functionality of the HLSC-EVs on the reduction of CSC invasion and increase of apoptosis, and was able to potentiate those effects by loading the anti-tumor miR-145. This was not specific for miR-145, as results were confirmed with miR-429. As expected, miR-145 alone, which was effective per se, was inactive after RNase treatment, at variance with coincubated EVs. This result excludes the possible additive effect of contaminating free miR-145, isolated as aggregates [17], on the effect of coincubated EVs on rCSCs. In addition, miR-145 showed a stable RNAse insensitive binding to the EV surface, as shown by super resolution microscopy and by the maintenance of miR transfer to target cells and of functional effects after RNAse treatment. The observed protection of surface-bound microRNA was likely due to the presence of RNA-binding proteins able to protect bound miRNAs. In particular, we identified the expression on the HLSC-EV surface of ANXA2, already described to play an active role in miRNA-loading in EVs [18]. This RNA binding protein was previously reported on human pancreatic cancer EV surface [19]. The involvement of surface RNA-binding proteins in miRNA loading and protection was confirmed by the loss of their activity after trypsin treatment.

At present, preclinical studies on oligonucleotide administration for cancer treatments mainly utilize EVs as a delivery system, obtaining them from therapeutically irrelevant engineered cells [25]. For instance, breast tumor cells were engineered with tumor suppressor miR-134 and let-7 and the deriving EVs were shown to display potent anti-tumor effects [26,27]. Similarly, 293 T cells were successfully transfected with siRNAs against c-Met, or with the anti-miR214 oligonucleotide to obtain anti-tumor EVs [28,29]. The advantages of this method are the low cost and the highly efficient EV loading. Furthermore, EVs have been directly electroporated with desired miRNAs. For instance, HepG2 cell-derived EVs were electroporated with miR-26 [30] and plasma-derived EVs with miR-31 and miR-451a [10] to gain anti-tumor activity. Similarly, fibroblast-derived EVs were engineered with siRNA or shRNA, specific to the oncogenic KrasG12D, gaining strong anti-tumor effect in models of pancreatic cancer [31]. Indeed, electroporated MSC-EVs with KrasG12D siRNA are currently being tested in a clinical trial (NCT03608631) on metastatic pancreatic ductal adenocarcinoma patients harboring a KrasG12D mutation. However, in all studies, the effect of naïve EVs was not required. This also applies in studies using electroporated MSC-EVs, in which the effect of naïve MSC-EVs was negligible [32,33]. Considering the multitude of active cargoes within EVs, it is likely that both cell transfection and direct electroporation may alter their functionality.

The protocol we propose here has the advantage of maintaining the endogenous property of EVs while adding the desired effect of the oligonucleotide therapeutic. This could be of application not only in cancer therapy, but also in regenerative medicine, considering the potent healing effect of EVs from MSCs and other stem cell types. In previous experiments, we failed to increase the repairing effect of MSC-EVs using miRNA loading obtained by MSC transfection, being the advantage only related to a lowering of the effective dose [34]. At the same time, it is at present unknown whether surface miRNA binding might be stable during the EV circulation in vivo. Another possible disadvantage of our protocol is the lower amount of miRNA linked to the EV surface with respect to that loaded by electroporation [10]. However, it has been recently shown that coincubation of ineffective serum EVs with miRNA was sufficient to promote angiogenesis in vitro and in vivo, suggesting that this method of EV engineering could be applied for autologous therapy (Tapparo M. et al., manuscript under revision).

In conclusion, here, we report a protocol of miRNA loading to engineer EVs with therapeutically active miRNAs without perturbing their cargo and innate characteristics. This protocol could be of interest for direct engineering of stem cell-EVs and exploits the presence of RNA binding proteins on EV surface. Further studies will be required to assess the miRNA stability and delivery by miRNA co-incubated EVs in in vivo settings.

## Data Availability

All data is available in the manuscript or the supplementary materials.

## Supplementary Materials

The following are available online at https://www.mdpi.com/1999-4923/13/1/76/s1, Figure S1: Comparison of electroporation and coincubation EV protocol on rCSC apoptosis and miR transfer. A: Percentage of apoptotic rCSCs treated with naïve HLSC-EVs (EV), or with HLSC-EVs either electroporated (EV-elettr) or coincubated (EV-coinc) with a scrambled sequence. Results are mean ± SD of three independent experiments. A-Nova was performed: * = *p* < 0.05 vs. untreated rCSCs (CTL). B: Real time analysis of miR-200a and miR-200b levels in rCSCs treated for 24 h with naïve HLSC-EVs (EV), or with HLSC-EVs either electroporated (EV-elettr), or coincubated (EV-coinc) with a scrambled sequence. Data are expressed as Relative Quantification (RQ) normalized to untreated cells (CTL) and to RNU6B.
