# Supplementary material for: Coincubation as miR-Loading Strategy to Improve the Anti-Tumor Effect of Stem Cell-Derived EVs
Source: Pharmaceutics. 2021 Jan 8;13(1):76. doi: 10.3390/pharmaceutics13010076 (PMC7826638; doi:10.3390/pharmaceutics13010076)
Supplement: Supplementary file 1 [file pharmaceutics-13-00076-s001.pdf]

pharmaceutics

Supplementary Materials: Coincubation as miR-Loading Strategy to Improve the Anti-Tumor Effect of Stem Cell-Derived EVs

Alessia Brossa, Marta Tapparo, Valentina Fonsato, Elli Papadimitriou, Michela Delena, Giovanni Camussi and Benedetta Bussolati

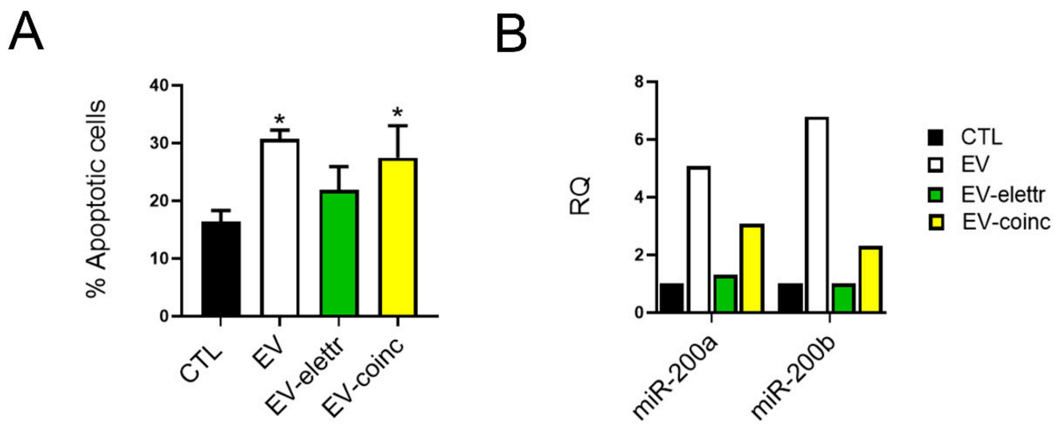

Figure S1. Comparison of electroporation and coincubation EV protocol on rCSC apoptosis and miR transfer. **A**: Percentage of apoptotic rCSCs treated with naïve HLSC-EVs (EV), or with HLSC-EVs either electroporated (EV-elettr) or coincubated (EV-coinc) with a scrambled sequence. Results are mean ± SD of three independent experiments. A-Nova was performed: * = $p<0.05$ vs untreated rCSCs (CTL). **B**: Real time analysis of miR-200a and miR-200b levels in rCSCs treated for 24h with naïve HLSC-EVs (EV), or with HLSC-EVs either electroporated (EV-elettr), or coincubated (EV-coinc) with a scrambled sequence. Data are expressed as Relative Quantification (RQ) normalized to untreated cells (CTL) and to RNU6B.
